# Mixed Matrix Membranes Composed of Graphene-Based Derivatives as Additives in PVAm for CO_2_ Capture

**DOI:** 10.1021/acsaenm.5c00316

**Published:** 2025-08-06

**Authors:** James Baker, Cristina Perinu, Maria Psarrou, Sigmund Mordal Lucasen, Victor Kusuma, Patrick F. Muldoon, Akrivi Asimakopoulou, David Hopkinson, Solon P. Economopoulos

**Affiliations:** † 17213 U.S. Department of Energy, National Energy Technology Laboratory, 626 Cochrans Mill Rd, Pittsburgh, Pennsylvania 15236, United States; ‡ NETL Support Contractor, 626 Cochran Mill Rd, Pittsburgh, Pennsylvania 15236, United States; § Department of Chemistry, 8018 Norwegian University of Science and Technology, 7491 Trondheim, Norway; ∥ Advanced Renewable Technologies & Environmental Materials in Integrated Systems, ARTEMIS, Chemical Process and Energy Resources Institute CPERI, Centre for Research and Technology Hellas CERTH, Thermi, 57001 Thessaloniki, Greece

**Keywords:** carbon capture, graphene, graphene oxide, mixed matrix membranes, graphene functionalization

## Abstract

Graphene oxide (GO) mixed matrix membranes (MMMs) that employ facilitated transport of carbon dioxide were prepared and tested for use in postcombustion carbon capture. Two graphene hybrids were synthesized using exfoliated graphene (G) and GO as a basis and were then appended with triethylene glycol (TEG) and *N*-(2-hydroxyethyl)­ethylenediamine (EDAOH) functional groups, respectively. Unfunctionalized GO nanoparticles were commercially obtained for comparison to the synthesized nanoparticles. The three additives were tested as nanofillers with loadings of 0.5 wt % and, in one case, 1 wt % in polyvinylamine (PVAm) matrices for CO_2_ and N_2_ gas permeability using humidified mixed gas. MMMs using G-TEG filler particles resulted in improved CO_2_/N_2_ selectivity, while GO-EDAOH fillers improved both the CO_2_ permeability and the CO_2_/N_2_ selectivity compared to neat PVAm. Unfunctionalized GO fillers resulted in no significant change in gas transport properties. Mechanical properties were also tested. The addition of GO or GO-EDAOH filler particles resulted in improvements in storage modulus as well as higher glass transition temperature, while G-TEG filler particles yielded a less significant change compared to neat PVAm.

## Graphical abstract

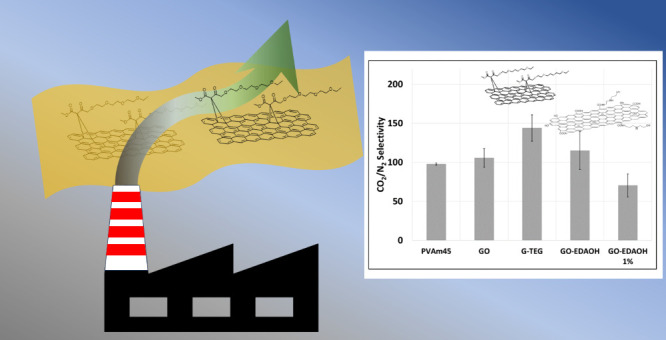

## 1 Introduction

As the atmospheric carbon dioxide level continues to rise, the need for technological solutions for large-scale industrial point source emissions like steel manufacturing, cement production, and electric power production is becoming increasingly urgent.^1^ In applications where a complete process redesign might otherwise be needed to reduce CO_2_ emissions, retrofitting the existing infrastructure with carbon capture technology is a pragmatic interim tool to greatly reduce CO_2_ emissions until new technologies can take its place.^2,3^ Membrane-based carbon capture is particularly attractive for retrofit applications due to its simple operation with no circulating media, minimal disruption to plant process design, and small footprint.^4^ However, the cost of capture is highly correlated with the performance of membrane materials, and this has led to an ongoing pursuit for ever higher performing materials by membrane developers.^5,6^

Postcombustion industrial point sources have exceptionally high volumes of CO_2_, thereby demanding membranes with high gas permeance to reduce the required membrane area and minimize the cost of capture.^5^ To improve the product purity, membranes should also have high CO_2_/N_2_ gas pair selectivity. These two targets are at odds with each other because of the well-known trade-off between gas permeability and selectivity that is inherent to polymer membranes that employ solution-diffusion as their primary mechanism for gas separation.^7,8^ On the other hand, facilitated transport membranes (FTMs) make use of active carrier sites that provide enhanced diffusion of CO_2_ and are therefore able to exceed the so-called Robeson upper bound.^9,10^ Polyvinylamine (**PVAm**) is one FTM polymer that has been widely reported for this purpose, making use of both fixed site carriers and mobile carriers for further improvements in the CO_2_ permeability and CO_2_/N_2_ selectivity. This is particularly attractive for carbon capture applications, but even FTMs can suffer from issues like poor mechanical stability and reduced gas permeability performance with decreasing film thickness.^11,12^

The addition of targeted filler particles can further enhance the performance properties of the FTMs. Exfoliated graphene and graphene oxide (**GO**) have been demonstrated as excellent platforms for filler particles in thin films because of their two-dimensional geometry and high aspect ratio. The use of carbon filler particles can lead to not only gas separation performance improvements but also an increase in mechanical stability.^13^ As with other types of mixed matrix membranes (MMMs), however, incompatibility between the matrix and filler can also lead to reduced membrane performance.

**PVAm** is the most studied amine-containing polymer for CO_2_ separation facilitated transport membranes due to its high density of amine sites to act as CO_2_ carriers, ease of synthesis from low-cost starting materials, and excellent film forming properties due to its ability to be synthesized in very high *M*_w_ (>5,000,000 g/mol).^14,15^ FTMs enhance the permeability and selectivity of the target solute, CO_2_ in this case, by a reversible reaction between carrier sites contained in the membrane and the solute. Due to the carrier saturation effect, permeability and selectivity for the solute increase as feed concentration decreases.^16,17^ This makes FTMs well suited to applications such as carbon capture from natural gas flue gas, where the CO_2_ concentrations in flue gas are only 4–5%. In **PVAm** the carrier sites are aliphatic amines that reversibly react with CO_2_ in the presence of water to form ammonium cations and carbamate or bicarbonate anions (Scheme 1).

**Scheme 1:**
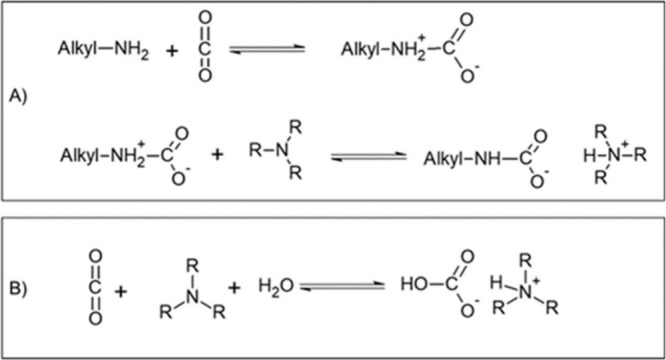
Reversible Reaction of CO_2_ with Amine Carriers in an FTM to Form A) Ammonium–Carbamate or B) Ammonium–Bicarbonate Ion Pairs

Here we present **PVAm** FTMs with chemically tailored graphene and graphene oxide filler particles for enhanced interfacial compatibility and CO_2_/N_2_ separation properties. For **PVAm** membranes, filler particles must be dispersible in water or water/alcohol mixtures, as these are the most effective solvents for **PVAm**. To accomplish this, we chose to append triethylene glycol (**TEG**) moieties with exfoliated graphene, as ether oxygen groups are known for enhancing gas separation in membrane materials. **TEG** has the added advantage of improving the dispersibility of graphene particles in polar solvents. By the same token, we also chose diamine structures to enrich the desirable aqueous solubility of graphene oxide with improved gas transport characteristics and selectivity from the amine group decoration. The functionalization followed established routes to synthesize novel compounds with custom-modified malonates with the specific aim not only to facilitate miscibility in **PVAm** but also to promote scalability over milligram scale for facile and repeatable testing of the MMM. The latter is very important since the application of functionalized graphene derivatives is challenging due to the fact that high-quality graphene is still difficult to obtain in both high quality and quantity. Therefore, GO and liquid-exfoliation-produced graphene are the more advantageous choices for applications, with each option possessing different drawbacks depending on the application. In MMM, specifically, the chemistry mostly focuses on producing uniform, reproducible composites with scalable results, rather than exotic decorations.^18–21^

By optimizing both the **PVAm** polymer and the chemical functional groups appended to exfoliated graphene, we can demonstrate an MMM that exceeds both the Robeson upper bound and the performance of commercially available membrane materials. Herein, we report synthetic procedures for decorating graphene with triethylene glycol, and graphene oxide with a diamine-containing functional group, as well as the membrane properties of MMMs incorporated with these filler particles.

## 2 Results and Discussion

In order to decorate exfoliated graphene with the triethylene glycol moiety, the cyclopropanation Bingel–Hirsch reaction scheme was followed.^22,23^ To allow for this decoration, the malonate moiety was attached onto the hydroxyl end-group of triethylene glycol monoethyl ether. The synthesis was similar to other malonates reported previously^24^ and involves a standard alcohol–acyl chloride reaction. The yield of the oily product was 85%. The synthesized graphene triethylene glycol hybrid (**G-TEG**) was prepared according to similar procedures previously reported by our group.^25^ The graphene analogues were exfoliated in NMP by using a tip sonicator and subsequently functionalized under microwave irradiation. The experimental parameters for various attempts are summarized in Table 1 and the synthetic process is depicted in Scheme 2. All of the samples used to characterize the hybrids and fabricate the **PVAm/G-TEG** mixed matrix membranes are from a statistical stock mixture containing all of the successful reactions that are listed in Table 1.

**Table 1:** Various Synthetic Parameters Used to Produce the Graphene Nanohybrids of the Triethylene Glycol-Substituted Malonate **G-TEG** Using the Microwave-Assisted Cyclopropanation Reaction

Malonate (mg) (mmol)	DBU (mL)	CBr_4_ (mg)	Graphene in NMP (mL)	Temp (°C)	Wattage	Time (min)
200 (0.68)	0.8	550	3	130	40	40
180 (0.61)	0.8	590	3	130	40	40
200 (0.68)	0.8	515	3	130	40	40
200 (0.68)	0.8	405	3	130	40	40
200 (0.68)	0.8	515	3	130	40	30
200 (0.68)	0.8	500	4	130	40	30
75 (0.26)	0.4	245	10	130	70	40

**Scheme 2:**
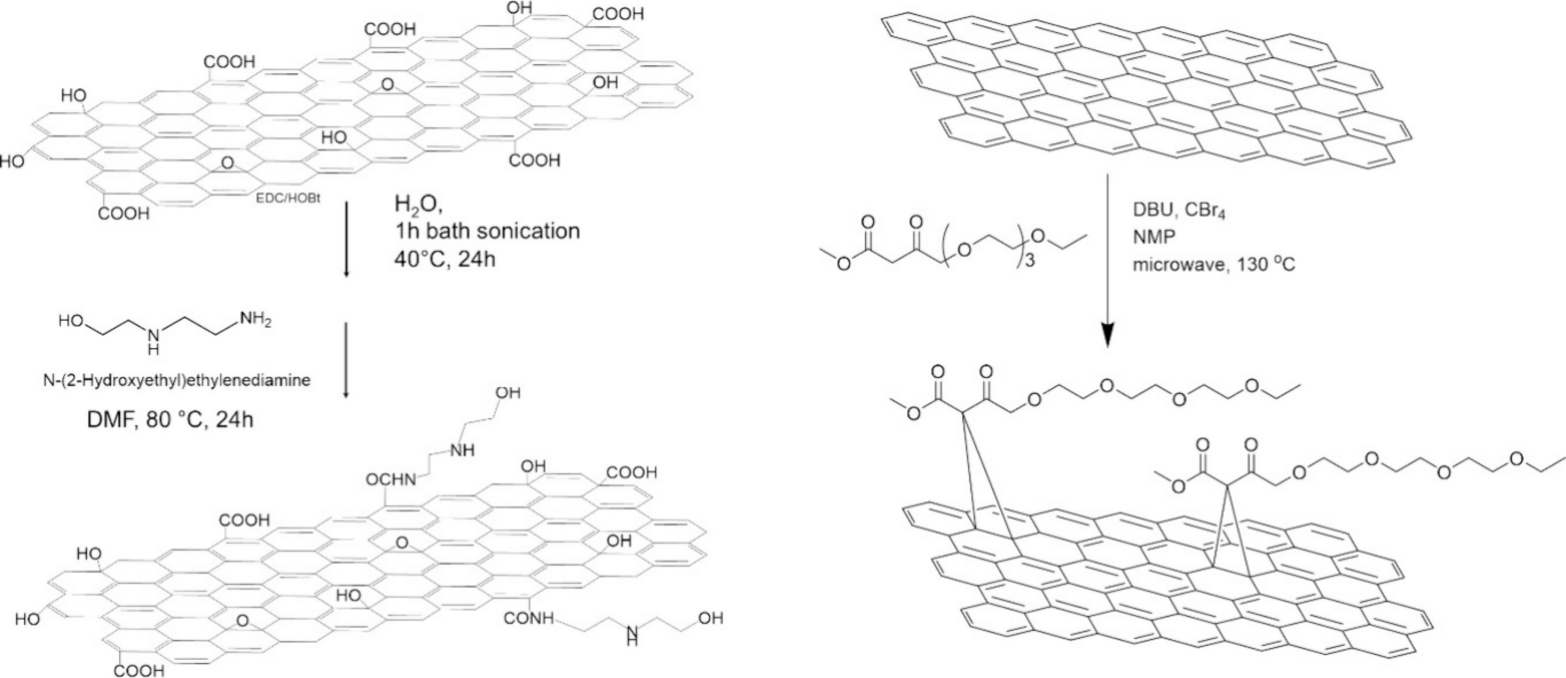
Structures of the Synthesized Graphene Hybrids with the **GO**-Analogue **GO-EDAOH** (Left) and the Exfoliated Graphene-Analogue **G-TEG** (Right)

All of the synthesized **G-TEG** hybrids showed good dispersibility in CH_2_Cl_2_, THF, etc. Conversely the **G-TEG** hybrid displays poor dispersibility in MeOH, hexane, and water. The material was stored dispersed in dichloromethane (DCM). Interestingly, the experiment that used the least amount of malonate, CBr_4_, and DBU (0.26 mmol, 245 mg, and 0.4 mL, respectively) has shown equal or even marginally improved functionalization, as evidenced by Raman spectroscopy. This indicates that this reaction is rather efficient and can be optimized further to produce larger quantities of the targeted graphene hybrid.

For the graphene oxide (**GO**) analogues a procedure similar to the one described by Ge et al. was employed^26^ for the preparation of **GO** starting material. The **GO** was exfoliated in water using tip sonication prior to use, and the resulting suspension was used for all subsequent syntheses. The **GO** was activated using 1-ethyl-3-(3-(dimethylamino)­propyl) carbodiimide (EDC) and 1-hydroxybenzotriazole hydrate (HOBt), and after stirring for 24 h at 40 °C, the reaction mixture was treated to remove unreacted **GO**. The product was reacted with *N*-(2-hydroxyethyl)­ethylenediamine (**EDAOH**) in DMF to afford **GO-EDAOH**.

### 2.1 Spectroscopic Characterization

The synthesized hybrids were characterized via microRaman spectroscopy to verify the covalent attachment of the triethylene glycol moiety to exfoliated graphene. In the case of the **GO**-adduct, Raman spectroscopy is used to verify the minimal spectroscopic change, denoting that functionalization did not take place on the graphitic backbone but rather on the carboxylic end groups.

From the spectroscopic analysis, Raman spectroscopy was used to verify the chemical modification of graphene in the case of **G-TEG**. Representative spectra are shown in Figure 1. Comparing the spectrum of exfoliated graphene in NMP, an *I*_D_/*I*_G_ ratio of 0.14 is calculated, while chemical modification using the Bingel–Hirsch cyclopropanation reaction conditions yields a *I*_D_/*I*_G_ ratio of 0.62, denoting successful incorporation of the malonate onto the graphitic backbone through the introduction of sp^3^ defects. In the case of **GO** analogues (Figure 1 right) the *I*_D_/*I*_G_ ratio for the exfoliated **GO** is 0.91. Upon formation of the peptide bond, the *I*_D_/*I*_G_ ratio increases to 1.07. It is expected that no additional defects are to be formed using this chemical functionalization since all reaction centers should be on the carboxylic defects already present on **GO**. However, small deviations are a common occurrence and are attributed to NH_2_ terminal groups forming covalent bonds to the graphitic backbone.^26^

**Figure 1:**
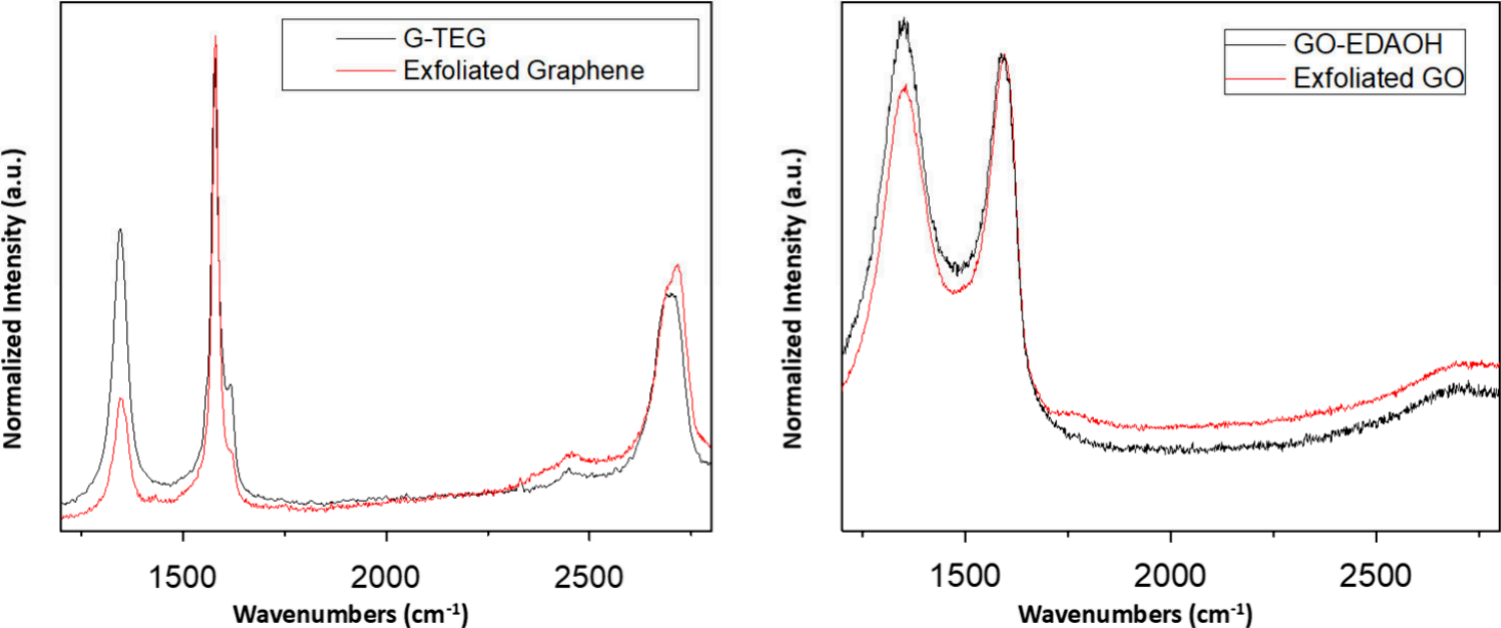
Representative microRaman spectra of the synthesized hybrids of **G-TEG** (left) and **GO-EDAOH** (right). Excitation: 532 nm.

### 2.2 Thermogravimetric Analysis

Thermogravimetric analysis of the materials is depicted in Figure 2. TGA is used to determine the degree of functionalization of the graphene analogue by assuming that the losses due to the heating of the hybrid are attributed solely to the organic moiety,^27,28^ since graphene is thermally stable up to 2000 °C. TGA was performed to verify the organic loading of each synthesized hybrid. Representative thermograms are shown in Figure 2.

**Figure 2:**
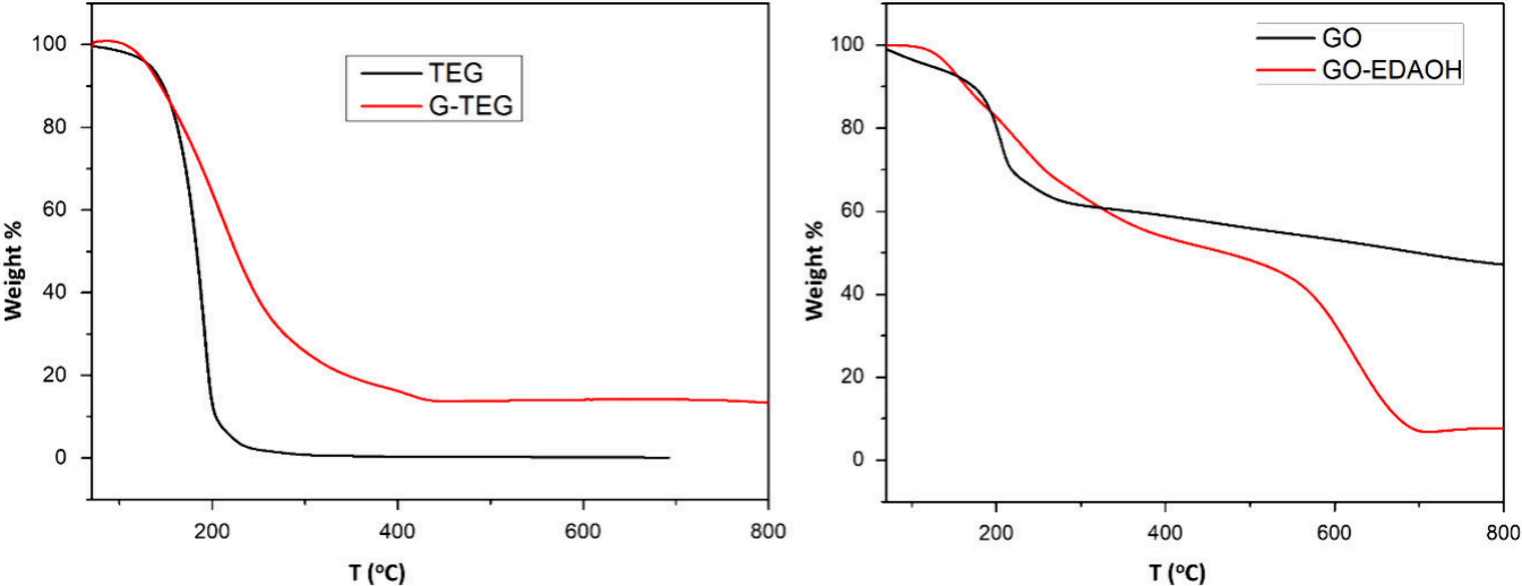
Typical thermograms of **G-TEG** (left) and **GO-EDAOH** (right) using TGA under a N_2_ atmosphere.

From the thermogram of the **G-TEG** we can see a sharp weight loss at around 208 °C, which can be attributed to the **TEG** moiety, which is also present in Figure 2. The **TEG** decomposition is observed at around 192 °C. There is an additional small step reduction in weight at 412 °C, which can be linked to the partial transformation of sp^3^- to sp^2^-hybridized carbons. The weight loss attributed to the decomposition of the TEG groups is observable until ∼280 °C and is responsible for close to 71% of the total weight loss of the sample. Based on this, the number of organic groups per number of carbons in the graphitic lattice is estimated at 1 organic addend group for every ∼10 carbon atoms in the graphene plane. Although this is a rough estimate and subject to various factors, such as organic addend size, reaction conditions, etc., this value is excellent and shows that the small **TEG**-malonate is an ideal candidate for highly decorated graphene hybrids.

The thermogram for the **GO-EDAOH** analogue shows several interesting points. For the GO starting material, at <400 °C we observe a ∼38% drop, which is attributed to decomposition of various functional groups and evaporation of residual solvent trapped in the nanosheets. There is an additional ∼10% weight loss from 400 to 600 °C, which is linked to the healing of defects, reverting the sp^3^-hybridized carbon atoms back to sp^2^ hybridization. Once functionalization using peptide chemistry conditions has taken place, the thermogram shows a roughly 50% weight loss taking place below 500 °C. Software analysis reveals that this drop consists of at least 2 different steps in weight loss due to decomposition of the different organic groups. A 16% weight loss occurs at around 156 °C, and a 34% weight loss occurs at around 221 °C. Thermogravimetric analysis of the commercially available **EDAOH** (not shown) reveals that the diamine decomposed completely at around ∼140 °C, and therefore the first weight loss should be attributed to this organic moiety loading. This analysis results in a highly functionalized hybrid with roughly 1 **EDAOH** group for every 45 carbon atoms. As mentioned in the Raman spectra discussion, there is **EDAOH** loading via NH_2_ covalent attachment along with the peptide bond attachment, which may explain such a high degree of functionalization. Finally, there is an additional 44% weight loss taking place above 600 °C, which can be linked to further decomposition of the hybrid material.

### 2.3 Preparation of Nanofiller Suspensions

Since **PVAm** only dissolves in water, dispersions of the nanofillers in a water-miscible solvent were needed. Initially, benzyl alcohol (BzOH) was tested since it is somewhat miscible with water (4 g/100 mL H_2_O) and the pi–pi interactions between the solvent and nanofiller may help to disperse the fillers. However, when the BzOH suspensions were added to a **PVAm** aqueous solution, an opaque-gray suspension resulted, indicating partial solvent immiscibility (Figure S1). Additionally, the high boiling point of BzOH ensured that some of this solvent would remain in the nanocomposite films even after drying, leading to films with an oily texture. To counter this, a mixture of EtOH and water (1:1) was used instead and proved to be adequate to disperse the nanofillers. Sedimentation of the fillers still occurred upon standing overnight in this solvent; therefore, the suspension was freshly sonicated for several minutes immediately prior to adding to the **PVAm** solution to minimize the presence of aggregates. Stock solutions of the nanofiller in solvent were generally 0.5 mg/mL in concentration (Figure S2). **GO-EDAOH** was a unique case in that it did not disperse well in EtOH, water, or mixtures of the two. MeOH proved to be the most effective water-miscible solvent for dispersing **GO-EDAOH**. A more dilute (0.2 mg/mL) suspension with **GO-EDAOH** was prepared, since this filler exhibited a greater propensity to form aggregates. Once the nanofiller/solvent was added to the **PVAm** solution at the required concentration, the rate of aggregation and sedimentation of the fillers were greatly reduced. This is likely due to the reduced concentration of nanofiller in the polymer solution (approximately 0.045 mg/mL), the higher viscosity of the polymer solution, and the hydrogen-bonding interactions between the amines of **PVAm** and the polar functionalities on the surface of the fillers. The difference in concentration is depicted in Figure S3.

Addition of the nanofiller stock solution to the **PVAm** aqueous solution required a specific procedure to prevent the aggregation of the fillers at the top of the solution. If the nanofiller/alcohol solution was simply added dropwise to the **PVAm** solution, there was a tendency for the nanofiller to float to the surface and form a film at the surface of the solution. The filler in this film layer would become strongly aggregated and could not be uniformly dispersed. Sonication of the **PVAm**/nanofiller solutions to disperse the filler was not an option since sonication led to chain scission of the **PVAm** chains. Chain scission is undesirable, since lowering the **PVAm** molecular weight could weaken the resulting films and may affect the gas permeation performance. Therefore, the nanofiller suspensions needed to be added to the **PVAm** solution in a way that limited phase separation. The most effective method was to rapidly stir the **PVAm** solution and add the nanofiller suspensions via pipet by immersing in the solution such that the pipet tip was just above the stir bar and then adding the solution dropwise slowly while allowing sufficient time for the nanofiller to disperse between each addition.

### 2.4 Polymer Films

**PVAm** was the matrix polymer for our facilitated transport membranes. **PVAm** was synthesized by first polymerizing *N*-vinylformamide to prepare poly­(*N*-vinylformamide) (PNVf), followed by acid hydrolysis to yield a random copolymer of NVf and PVAm repeat units (Figure S4). The **PVAm** content used for this project contained 45 mol % amines (55 mol % NVf), since previous research determined this amine content provides the best CO_2_ permeability and selectivity for **PVAm** membranes.^29^ Films of **PVAm** containing 0.5 wt % nanofiller were generally uniform in appearance, with a gray tint (Figure 3). **GO-EDAOH** was also tested at 1 wt % concentration, but significant formation of macroaggregates was observed in the film, indicating this concentration was too high to uniformly disperse in the polymer.

**Figure 3:**
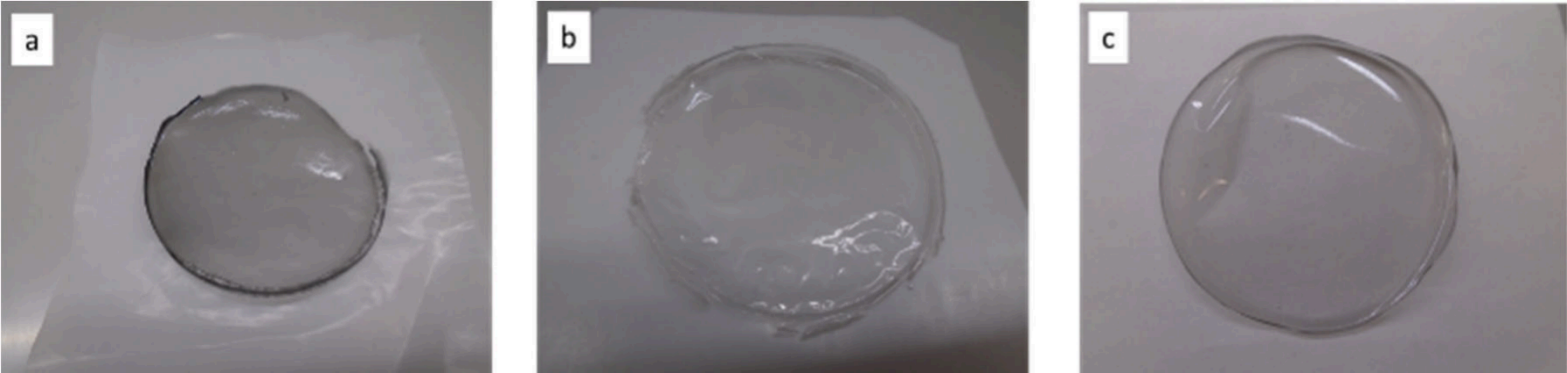
Bulk films (7 cm diameter, 33 μm thickness) of a) **PVAm**+ 0.5 wt % **GO**, b) **PVAm**+ 0.5 wt % **G-TEG**, and c) **PVAm**+ 0.5 wt % **GO-EDAOH**.

### 2.5 Gas Permeability

The CO_2_ permeability and CO_2_/N_2_ selectivity of the **PVAm** films were measured using simulated flue gas containing 4 wt % CO_2_ (balance N_2_) on a custom-built permeation system that allowed humidification of the feed and sweep gas streams.^30^ The water vessels and membrane module are contained within a temperature-controlled oven to prevent condensation of water in the gas lines. Water vapor is a constituent of most flue gas streams, and in the case of **PVAm** water vapor is beneficial since it swells the polymer, making it more gas permeable, and is also necessary for the facilitated transport reaction.^31^ Relative humidity for the permeation experiments was kept above 95% to simulate the high moisture content of the flue gas streams.

The gas permeation results are listed in Figure 4. A minimum of 3 coupons of each material with a mean thickness of 33 ± 3 μm were tested. Comparing films of similar thickness is important since the solute permeability and selectivity of FTMs vary with film thickness.^13,32–34^ For neat **PVAm45** containing no fillers, the average CO_2_ permeability was 880 barrer (1 barrer = 1 × 10^–10^ cm^3^(STP) cm/(cm^2^·s·cmHg)) with a CO_2_/N_2_ selectivity of 98. Adding 0.5 wt % commercial **GO** slightly reduced the permeability to 828 barrer with 105 selectivity. The **PVAm45** with 0.5 wt % **G-TEG** exhibited nearly the same permeability as **PVAm** at 866 barrer with an improved selectivity of 144. The films with 0.5 wt % **GO-EDAOH** exhibited improved permeability of 1135 barrer with 115 selectivity. Increasing the **GO-EDAOH** loading to 1 wt % diminished the permeability to 1020 barrer and had a negative impact on selectivity, reducing it to 70. From these results 0.5 wt % **GO-EDAOH** improved CO_2_ permeability by 28% compared to the pristine polymer sample, with a 15% improvement in selectivity. Samples with 0.5 wt % **G-TEG** improved CO_2_/N_2_ selectivity by 46% but did not yield any permeability improvements. For the given testing conditions, all the films tested fall above the Robeson upper bound for CO_2_/N_2_ separations (Figure S5)

**Figure 4:**
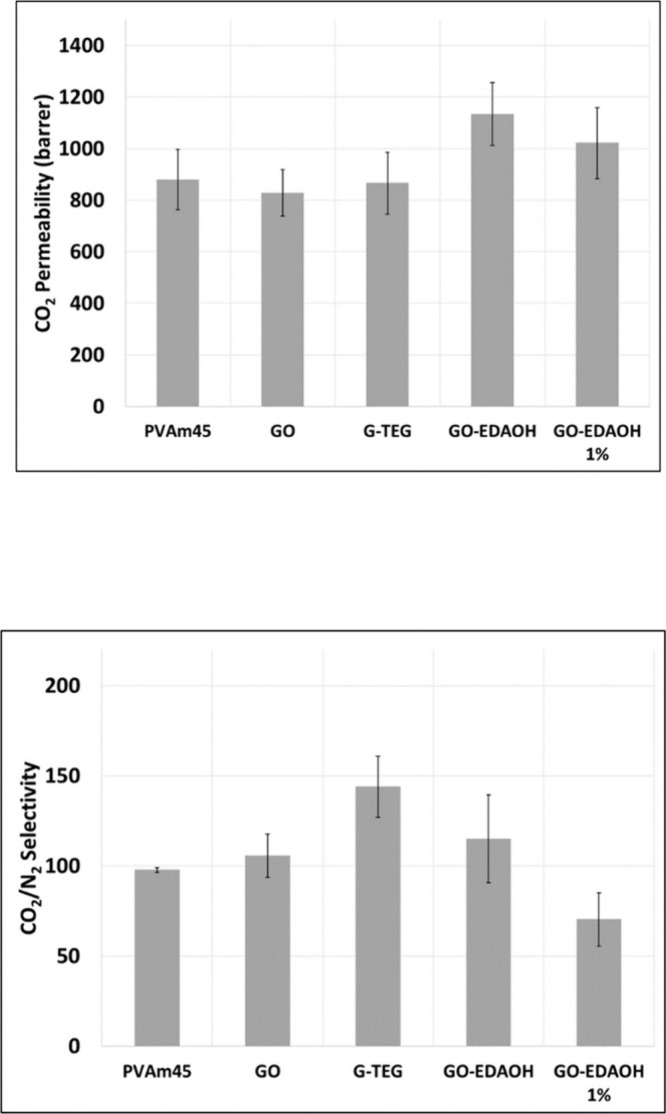
Comparison of the CO_2_ permeability (top) and CO_2_/N_2_ selectivity (bottom) for **PVAm** films containing 0.5 wt % nanofiller (unless otherwise noted). Permeation testing conditions: 60 °C, 95% relative humidity, feed gas 70 sccm 4/96 CO_2_/N_2_ at 1.5 ata, sweep gas 20 sccm He at 1.22 ata.

The addition of 2D filler particles will disrupt the chain packing of **PVAm** but can also introduce impermeable regions in the film. Both commercial **GO** and **G-TEG** resulted in films with CO_2_ permeabilities similar to that of the neat **PVAm**. However, addition of 0.5 wt % **GO-EDAOH** to the films led to an increase in CO_2_ permeability relative to neat **PVAm**. The surface functionalities on commercial **GO** are typically a mixture of hydroxyl, carboxylic acid, and epoxide groups.^35^ The carboxylic acid groups will interact strongly with the amines of **PVAm** through acid–base interactions,^36–38^ which may lead to a diminished mobility or densification of the polymer chains at the surface of the **GO**. In **GO-EDAOH** a portion of these carboxylic acid functionalities are converted to amides and the resulting **EDAOH** functional groups are terminated with a hydroxyl group. These will interact with the amines of **PVAm** through hydrogen bonding, but the interaction will be weaker than that in the case of the carboxylic acid groups. Similar interactions leading to altered CO_2_ permeability behavior have been demonstrated between polyimide and phenyl acetyl functionalized particles,^39^ and interactions have also been quantified by Fourier-transform infrared spectroscopy (FT-IR) for amino-functionalized particles with dibenzodioxane linkages in polymers of intrinsic microporosity (PIM-1).^40^ The epoxide groups on **GO** can also react with the amines on **PVAm** through epoxy-amine chemistry. This would lead to covalent attachment of **PVAm** to **GO**, again reducing the mobility of **PVAm** at the **GO** surface. For **GO-EDAOH**, the amines of **EDAOH** can react with the epoxides during the functionalization reaction, resulting in hydroxyl-functional groups on the surface of the nanofiller instead of epoxides. Attenuating the interaction strength between **GO** and **PVAm** by **EDAOH** prevents densification of **PVAm** at the filler–matrix interface. This type of filler–matrix interaction has been demonstrated previously using Monte Carlo molecular modeling, in which reduced interaction between polymer and particle resulted in a corresponding reduction of interfacial density of polymer chains.^41^ In the case of **G-TEG**, the TEG functionalization was adequate to make the normally hydrophobic graphene hydrophilic enough to disperse water/alcohol solvent for addition to the **PVAm** solution. The TEG functional group will interact less strongly with the amines of PVAm since it lacks the H-bonding donors present on **GO** and **GO-EDAOH**.

The improvements in CO_2_/N_2_ selectivity observed in films containing **GO**, **G-TEG**, and **GO-EDAOH** may be due to the 2D nanofillers creating a more circuitous path for the gas molecules to diffuse through the membrane.^42^ The apparent CO_2_ permeability and selectivity of FTMs increase as film thickness increases due to the facilitation factor increasing with film thickness.^13,32–34^ The facilitation factor is defined as the ratio of solute permeance via both facilitated transport and solution diffusion combined with that of solute diffusion only due to solution diffusion. An increase in the gas diffusion path length due to the impermeable nanofillers would have the effect of increasing the apparent thickness of the film. This is expected to increase the CO_2_ permeability without change to the N_2_ permeability, resulting in an increase in CO_2_/N_2_ selectivity. The 0.5 wt % loading of all 3 nanofillers led to increases in the selectivity compared to neat **PVAm**. The films containing 1 wt % **GO-EDAOH** exhibited macroaggregation of the nanofiller and had a rough surface. These large aggregates act as defects that can reduce selectivity. Addition of **G-TEG** nanofillers produced the largest improvement in the selectivity. The smaller particle size and weaker polymer–filler interaction for **G-TEG** may have caused it to disperse more uniformly in the composite films. The **G-TEG** nanofiller also possessed a large weight fraction (∼70 wt %) of TEG surface groups having CO_2_-philic ether oxygens that may contribute to increased CO_2_/N_2_ selectivity.

The combined selectivity and permeability performance of the produced membranes “doped” with functionalized carbon nanomaterials is on par and in many instances significantly improved compared to similar examples using graphene/GO/carbon nanotubes, reported in the literature.^34,43–45^ However, it is our belief that such comparisons hold little weight, as there are significant differentiations in experimental conditions, carrier gases, sample thicknesses, etc., making direct comparisons somewhat misleading. Even if the comparison can be favorable, we would like to focus on our significant effort to benchmark our samples with our synthesized pristine **PVAm**, holding all relevant parameters stable to truly exhibit relevant changes.

### 2.6 Physical Characterization

The nanofillers also provide mechanical reinforcement to the polymer film, which reduces the amount of polymer penetration into a porous support of a thin film composite membrane. In this configuration, a thin film of the membrane selective layer is cast onto a highly porous support layer to maximize the gas permeance of the composite membrane. Intrusion of the selective layer into the pores of the porous support results in increased mass transfer resistance and, therefore, reduced gas permeability. **PVAm** is a very hydrophilic polymer, and high humidity is used during the gas permeation experiments since this improves both the CO_2_ permeability and selectivity.^46^ High humidity swells **PVAm** and makes the polymer film very soft, which can cause the polymer to be pushed into the support pores by transmembrane pressure. Nanofillers, such as carbon nanotubes, have been used to reinforce **PVAm** to mitigate this issue.^47–49^ The addition of nanofillers to **PVAm** in this work also resulted in the reinforcement of the polymer films.

Humid dynamic mechanical analysis (DMA-RH) experiments were conducted to quantify the effect of the nanofillers on the film mechanical properties. The relative humidity (RH) was controlled at 50% RH during the experiments. Testing the films under a humid atmosphere provides a more representative value since the **PVAm** membranes are used in the rubbery, hydrated state during gas separations, not in the dry glassy state. 50% RH was selected since at higher RH the samples were already above the glass transition temperature (*T*_g_) of 5 °C, which is the lowest temperature the instrument could provide.

The storage modulus of the films containing 0.5 wt % of filler particles was measured at 10 °C under 50% RH (Figure 5). Both **GO-EDAOH** and **GO** led to significant increases in the modulus of the films. Neat **PVAm** had a modulus of 2400 MPa. This increased to 7300 MPa for the films containing **GO-EDAOH** and 6360 MPa for films with **GO**. The **G-TEG** filler led to less reinforcement with a storage modulus of 2420 MPa. The high weight fraction of organic grafting and smaller particle size for **G-TEG** compared to the **GO** materials may have reduced the impact of this filler on the modulus. Since **G-TEG** was approximately 70% TEG by mass, the amount of graphitic carbon loaded into the films containing 0.5 wt % **G-TEG** will be less than for films containing 0.5 wt % **GO-EDAOH**, which was only 16 wt % organic functionalities by TGA analysis. Additionally, the stronger interactions between the surface functionalities of **GO** and **GO-EDAOH** contributed to these fillers providing better mechanical reinforcement. The storage moduli trend was also observed at 30 °C where all the polymers would be in the rubbery state (Figure S6). The *T*_g_ of the composite films under 50% RH was also measured (Figure 6). The mean *T*_g_ for neat **PVAm** was 13.0 °C under 50% RH. This increased to 23.4 °C for the film with 0.5 wt % **GO**. The *T*_g_ was 25.1 °C for the films containing 0.5 wt % **GO-EDAOH**. **G-TEG** had less effect, with a *T*_g_ of 16.8 °C at 0.5 wt % loading. The trend for an increase in *T*_g_ for the films containing the **GO**-based fillers is consistent with storage modulus trends, where these fillers add rigidity to the polymer film and reduce the polymer chain mobility, even when the polymer is under humidity.

**Figure 5:**
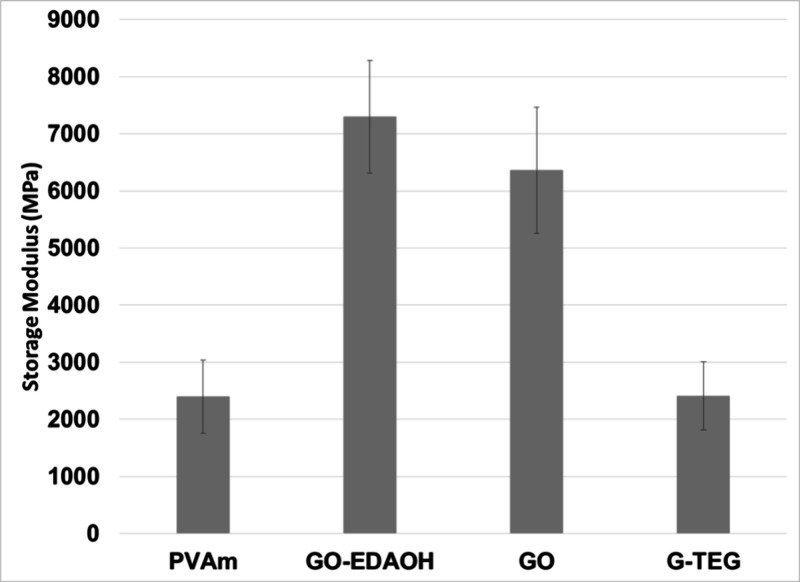
Storage modulus of **PVAm** films, both neat and containing 0.5 wt % filler particles, at 10 °C under 50% relative humidity as measured by DMA-RH.

**Figure 6:**
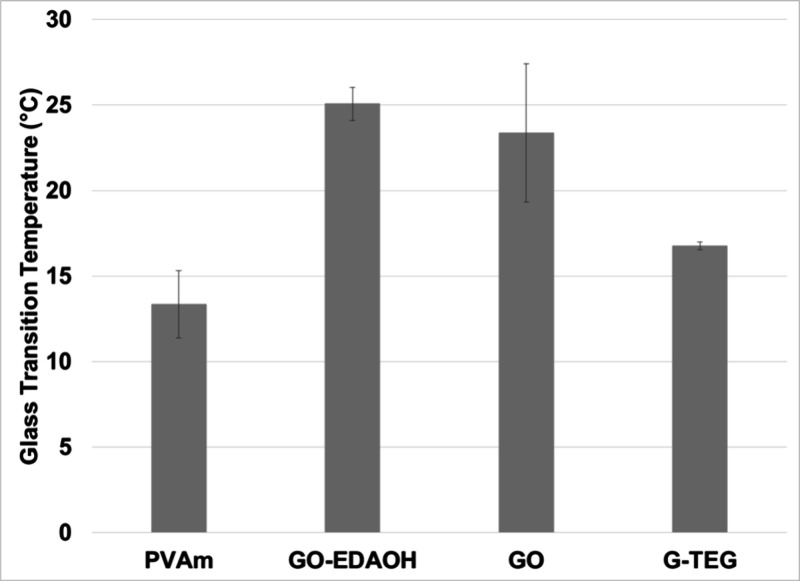
Glass transition temperature for **PVAm** films, both neat and containing 0.5 wt % filler particles, under 50% relative humidity as measured by DMA-RH.

Scanning electron microscopy (SEM) was used to examine the cross sections of freeze-fractured samples of the **PVAm** films (Figure 7). The neat **PVAm** film had a smooth fracture surface without any particles or fracture planes visible. The film containing commercial **GO** exhibited many fracture lines in the plane of the cross section. These may be points where sharp fractures occurred at the polymer–filler interface. The film containing **GO-EDAOH** was noticeably smoother than the one with a commercial **GO** filler. Finally, the fracture surface of the **PVAm**/**G-TEG** film had more texture, with fracture planes parallel to the surface of the film. In all 3 nanofilled polymer matrices, there are raised sections with sharp edges that may be attributed to the graphene/**GO** nanoparticles that are oriented parallel to the plane of the surface. The size of the embedded nanoparticles is on the order of a few microns. In none of the films are aggregates of the nanofillers visible, indicating good dispersion. There are significant differences between the 0.5% and 1% images of the composite (Figure 7c and 7e) with the higher-loading SEM image, showing considerable inconsistencies throughout the bulk of the layer. Aggregates of the nanofiller are visible in these images as regions with a different texture than the overall film. The SEM image is in good accord with our macroscopic evaluation of aggregate formation during the preparation of the sample.

**Figure 7:**
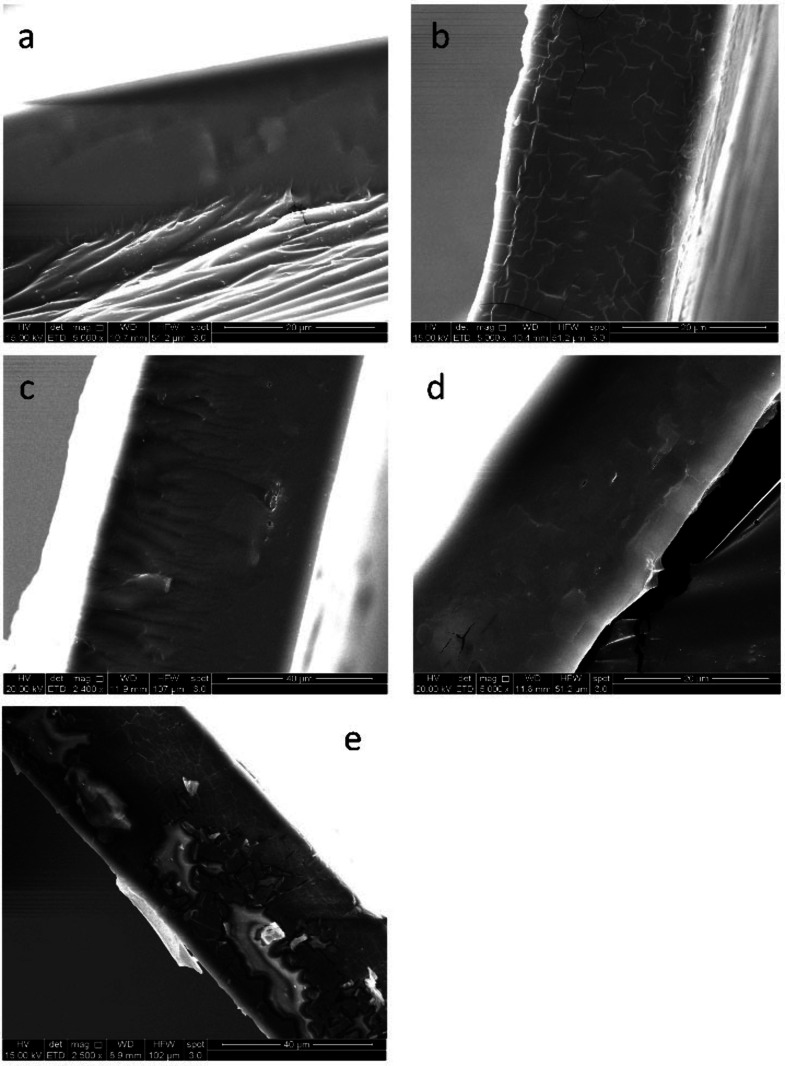
SEM cross section images of a) **PVAm**, b) **PVAm** + 0.5 wt % **GO**, c) **PVAm** + 0.5 wt % **GO-EDAOH**, d) **PVAm** + 0.5 wt % **G-TEG**, and e) **PVAm**+ 1 wt % **GO-EDAOH**.

## 3 Conclusion

Two functionalized graphene hybrids were synthesized based on exfoliated graphene and graphene oxide aiming to improve the miscibility and dispersibility of carbon nanoparticles in poly­(vinylamine) (PVAm) polymer matrices for CO_2_ capture applications. Mixed matrix membranes using the synthesized filler particles as well as commercially obtained graphene oxide filler particles were investigated for their gas transport and mechanical properties. The addition of 0.5 wt % **GO-EDAOH** to PVAm was found to disrupt polymer chain packing while having an attenuated interaction with amine groups, leading to an increase in CO_2_ permeability compared with neat PVAm. On the other hand, the addition of 0.5 wt % **G-TEG** filler particles led to the largest gains in CO_2_/N_2_ selectivity, likely due to its small particle size and high dispersibility in the matrix, leading to a more circuitous path for gas molecules to diffuse through the membrane. **GO-EDAOH** also resulted in a more modest increase in CO_2_/N_2_ selectivity at 0.5 wt % loading, but decreased selectivity at 1 wt % loading due to particle agglomeration. Notably, the two synthesized nanoparticles (**G-TEG** and **GO-EDAOH**) resulted in much greater gains in gas separation properties than commercially obtained **GO**. **GO** and **GO-EDAOH** filler particles resulted in significant mechanical reinforcement of PVAm films, in terms of both storage modulus and *T*_g_. The effect was less significant for **G-TEG** filler particles, likely due to the smaller particle size and high weight fraction of organic grafting.

## 4 Experimental Procedure

### 4.1 Materials

All chemical reagents and solvents were purchased via Merck and were used without further purification, unless otherwise stated. Column chromatography was performed on silica gel (Merck TLC-Kieselgel 60H, 15 lm). *n*-Octane (99%) was purchased from Oakwood Chemical (Estill, SC). *N*-Vinylformamide (NVf, 96%) was purchased from TCI Chemical (Montgomeryville, PA) and vacuum distilled at 85 °C/1 mmHg prior to use. Distilled NVf was stored in a refrigerator at 4–8 °C. Reagent ethanol (EtOH), hydrochloric acid (HCl, ACS grade, 12 M), methanol (MeOH), silver nitrate (AgNO_3_), sodium nitrate (NaNO_3_), and sorbitan monostearate (Span60) were purchased from Fisher Scientific and used as received. Anion exchange resin, Purolite A600OH form, was donated by Purolite LLC (King of Prussia, PA). Polyacrylamide calibration standards for gel permeation chromatography (GPC) were obtained from American Polymer Standards (Mentor, OH). Reverse osmosis water generated by an Aqua Solutions water purification system was used for all experiments and polymer solutions.

### 4.2 Instrumentation

Thermogravimetric analysis was performed on a TA Instruments TA Q55. All samples were measured in a platinum pan under N_2_ flow at 40 mL/min using the following set program: equilibration at 70 °C for 30 min, ramp to 800 °C at 10 °C/min, cool down to room temperature. MicroRaman spectroscopy was performed on a microRaman Renishaw spectrometer using the 532 nm excitation laser line. ^1^H NMR spectra were recorded on a Bruker Avance 500 instrument at 400 MHz. Deuterated solvents were used for homonuclear lock, and the signals are referenced to the deuterated solvent peaks. Ultrasonication processes for exfoliating graphene and producing the starting nanomaterial for all covalent reactions were conducted on a SONOPULS HD2070 tip-sonicator at ∼60 W output power, using NMP as the exfoliating agent. Microwave synthesis was conducted on a Biotage Initiator microwave reactor.

### 4.3 Synthesis of **GO-EDAOH**

In a typical experiment, exfoliated **GO** was placed in a 100 mL vial, followed by addition of ∼31.3 mg of 1-ethyl-3-(3-(dimethylamino)­propyl)­carbodiimide (EDC) and ∼35.6 mg of 1-hydroxy­benzotriazole hydrate (HOBt). After bath sonication for 1 h, the reaction mixture was stirred for 24 h at 40 °C. The resulting mixture was cooled to room temperature and centrifuged in order to remove the unreacted **GO**. The supernatant was discarded away, and the black solid precipitate was filtered, washed with ionized water and DMF, transferred to a round-bottom flask along with 25 mL of DMF, and bath sonicated for *t* = 1 h. To this dispersion was added 25 mL of *N*-(2-hydroxyethyl)­ethylenediamine (EDAOH), and the mixture was refluxed under N_2_ at 80 °C for 24 h. The reaction was stopped by addition of EtOH, and the reaction mixture was filtered and washed to remove the unreacted amine. The filtered product was centrifuged using MeOH, and the supernatant was stored and used for this study.

### 4.4 Synthesis of **TEG** Malonate

Triethylene glycol monoethyl ether (1.78 g, 10 mmol) and dry pyridine (0.98 g, excess) were added to an oven-dried, 100 mL round flask containing 50 mL of anhydrous CH_2_Cl_2_. The mixture was placed in an ice bath, and after a cooling period, ethyl malonyl chloride (1.5 g, 10 mmol) was then slowly added to the round flask via syringe over the course of 5 min. After addition, the reaction mixture was left in the ice bath for 1 h and subsequently allowed to react for 72 h at room temperature. After completion, the reaction was quenched with saturated NaCl solution (40 mL) and extracted using 4 × 100 mL of brine and 2 × 100 mL of distilled water. The organic layer was dried with MgSO_4_ and filtered, and a light amber tinted oily crude product was obtained after rotary evaporation. The crude product was purified by column chromatography, using acetonitrile as an eluent, yielding a bright yellow tinted oil (2.48 g, 8.5 mmol, 85% yield). ^1^H NMR (400 MHz, CDCl_3_) δ_H_: 4.30 (t, *J* = 4.64 Hz, 2H, H6), 4.20 (q, *J* = 7.09 Hz, 2H, H2), 3.72 (t, *J* = 4.98 Hz, 2H, H7), 3.66 (m, 6H, H8–10), 3.59 (t, *J* = 4.49 Hz, 2H, H11), 3.53 (q, *J* = 7.07 Hz, 2H, H12), 3.40 (s, 2H, H4), 1.28 (t, *J* = 7.30 Hz, 3H, H13), 1.21 (t, *J* = 7.17 Hz, 3H, H1). ^13^C NMR (100 MHz, CDCl_3_) δ_C_: 166.6 (C5), 166.5 (C3), 70.72, 70.61, 70.58 (C8–C10), 69.8 (C11), 68.9 (C12), 66.6 (C7), 64.6­(C6), 61.5 (C2), 41.5 (C4), 15.1 (C13), 14.1 (C1). IR (neat, cm^–1^) ν: 2865 (C–H stretch, alkane), 1730 (C=O stretch, ester), 1266 (C–O stretch, ester), 1106 (C–O stretch, ester), 1032 (C–O stretch, ester). HRMS (TOF MS ASAP+ *m*/*z*): 315.1425 (calculated: C_13_H_24_O_7_Na: 315.1420 [M + Na]).

### 4.5 Synthesis of **G-TEG**

In a typical experiment, CBr_4_, TEG malonate, exfoliated graphene, and DBU were added under nitrogen flow. The vial was then sealed and inserted into the microwave reactor. The reaction was run to completion by using the parameters described in Table 1. The temperature limit was set to 130 °C. Following completion, the reaction mixture was filtered and washed consecutively with DMF, MeOH, and CH_2_Cl_2_. The solid residue was collected by using CH_2_Cl_2_ and centrifuged to remove unreacted graphene. The supernatant was collected as an opaque black dispersion.

### 4.6 Synthesis of **PVAm**

#### Poly­(*n*-vinylformamide) (PNVf)

PNVf was synthesized in a modification of literature procedures^29,50,51^ as follows: *n*-octane (60 g) and Span60 (1.6 g) were added to a 500 mL two-neck round-bottom flask equipped with a condenser and overhead motor stirrer mixing at 300 rpm and heated at 50 °C to dissolve the Span60 while sparging with N_2_. After 15 min, a solution of NVf (10 g, 140 mmol) in 14 g of RO water was added to the flask and sparging continued for 1 h. A solution of AIBN (3 mg, 0.018 mmol) in 0.5 mL of toluene was added, and the temperature increased to 70 °C while stirring under N_2_ for 2 h. The resulting opaque white emulsion was slowly poured into 300 mL of EtOH heated to 65 °C to precipitate the polymer. The polymer was collected, dried, and redissolved in 150 mL of H_2_O while heating at 50 °C. Once the PNVf had fully dissolved, the temperature was increased to 65 °C and EtOH (600 mL) was slowly added to precipitate the polymer. The polymer was collected and dried to yield 6.2 g of white solid. GPC *M*_n_ = 2,050,000 g/mol, *M*_w_ = 11,200,000 g/mol.

#### Polyvinylamine (**PVAm**)

**PVAm** was synthesized by acid hydrolysis of PNVf.^50,52^ PNVf (5.1 g, 72 mmol) was dissolved in 250 mL of H_2_O at 55 °C. HCl (9 mL, 108 mmol) was added, and the temperature increased to 70 °C for 6 h. An aliquot (approximately 5 mL) was set aside for conductometric titration. The remaining **PVAm-**HCl was precipitated by pouring into EtOH (500 mL), and the solids were collected and dried in a vacuum oven overnight at 40 °C. The polymer was then redissolved in 350 mL of H_2_O and treated with Purolite A600OH form anion exchange resin until pH 11 with stirring at ambient temperature for 30 min to convert the amine hydrochlorides to deprotonated amines, after which the solution was vacuum filtered to remove the resin. The **PVAm** solution was stored at 4–8 °C to inhibit the further hydrolysis of the formamide units.

### 4.7 Preparation of Nanofiller Suspensions

Suspensions of the nanofillers were prepared as in the following example. **GO** powder (2 mg) was added to a glass vial, and any clumps in the powder were broken apart with a spatula. Solvent (4 mL, 1:1 H_2_O:EtOH) was added to the vial to prepare a 0.5 mg/mL suspension, the vial capped, and the suspension sonicated in a Branson 1510 sonicator bath for 30 min to disperse the **GO**. Since the suspensions settled out upon standing for long periods, the suspensions were sonicated for 5 min immediately prior to addition to the **PVAm** solutions.

### 4.8 Preparation of Nanocomposite Films

Polymer nanocomposite films were prepared as in the following example. A solution of 9.3 g of **PVAm** 0.95 wt % solution in H_2_O (88 mg of **PVAm**) was added to a glass vial. The vial was placed on a stirrer and stirred rapidly (900 rpm). A 2.2 g amount of a freshly sonicated 0.2 mg/mL suspension (0.44 mg **GO-EDAOH**) in MeOH was slowly added via a plastic pipet by immersing the pipet tip to just above the stir bar when dispensing the suspension to prevent the nanofiller from aggregating at the solution surface. The vial was capped, and the polymer solution was stirred for 1 h to fully mix. The **PVAm/GO-EDAOH** solution was poured into a PTFE mold and placed on a leveled plate in an oven at 30 °C under N_2_ for 2 days. Once the dry film had formed, it was carefully peeled from the mold. The average dry film thickness was 33 μm.

### 4.9 Gas Permeation Testing

Gas permeation testing coupons were cut from the polymer films with a circular die (1″ diameter) and placed on a Supor 100 poly­(ether sulfone) porous support (1″ diameter). The supported film was then placed on top of a stainless-steel frit (2 μm mesh) in a stainless-steel membrane testing module and sealed with a Viton O-ring (size 18). The effective film surface area tested was 2.85 cm^2^. Permeation tests were conducted on a custom-built isobaric (constant pressure) system with the membrane module and all humidified lines contained inside a temperature-controlled oven (Tenney T10, Thermal Products Solutions). A more thorough description and schematic of the system can be found in prior publications by our group.^30^ The feed gas was premixed 4% CO_2_/ balance N_2_ supplied by Butler Gas Company (McKees Rocks, PA) at 1.5 atm at a flow rate of 70 cm^3^/min. The sweep gas was Ar at 1.22 atm with a flow rate of 20 cm^3^/min. The feed and sweep streams were humidified by bubbling the gas through distilled water in stainless steel vessels inside the oven set at 60 °C. The permeate composition was measured using a PerkinElmer Clarus 500 gas chromatograph (GC). The permeate stream was dehumidified by passing through a Baldwin M425D thermoelectric chiller (PermaPure) before reaching the GC. The CO_2_/N_2_ selectivity from mixed gas permeation tests was determined using the following equation (eq 1), where *P*_CO2_ and *P*_N2_ are the measured CO_2_ and N_2_ permeances.

1 $$\alpha_{ij}=\frac{P_{CO2}}{P_{N2}}$$

### 4.10 Dynamic Mechanical Analysis Testing (DMA)

DMA testing was conducted on a TA Instruments Q800 DMA equipped with a relative humidity (DMA-RH) accessory. The samples were tested in multistrain mode using a film tension clamp with an oscillatory displacement of 20 μm at a frequency of 6 Hz. The relative humidity was set at 50%, and the temperature ramp rate was 1 °C/min after a 30 min equilibration at 5 °C. Typical sample size was 5.3 mm width and 10 mm gauge length with an average film thickness of 45 μm. The *T*_g_ was measured as the peak in the loss modulus curve. A minimum of 3 specimens of each sample were tested.

## Supplementary Material



## Notes

Images of polymer–graphene dispersions, additional storage moduli graph at 30 °C, chemical structure of **PVAm** used in this work, and Robeson plot for all **PVAm**-based bulk films (PDF)

## References

[1] Intergovernmental Panel on Climate, C. Climate Change 2022 – Impacts, Adaptation and Vulnerability: Working Group II Contribution to the Sixth Assessment Report of the Intergovernmental Panel on Climate Change; Cambridge University Press, 2023.

[2] Baker R. W., Freeman B., Kniep J., Huang Y. I., Merkel T. C. (2018). Co2 Capture
from Cement Plants and Steel Mills Using Membranes. Ind. Eng. Chem. Res..

[3] Scholes C. A., Ho M. T., Aguiar A. A., Wiley D. E., Stevens G. W., Kentish S. E. (2014). Membrane Gas Separation
Processes for Co2 Capture from
Cement Kiln Flue Gas. Int. J. Greenh. Gas Control.

[4] Han Y., Salim W., Chen K. K., Wu D., Ho W. S. W. (2019). Field
Trial of Spiral-Wound Facilitated Transport Membrane Module for Co2
Capture from Flue Gas. J. Membr. Sci..

[5] Merkel T. C., Lin H., Wei X., Baker R. (2010). Power Plant Post-Combustion Carbon
Dioxide Capture: An Opportunity for Membranes. J. Membr. Sci..

[6] Turi D. M., Ho M., Ferrari M. C., Chiesa P., Wiley D. E., Romano M. C. (2017). Co2 Capture
from Natural Gas Combined Cycles by Co2 Selective Membranes. Int. J. Greenh. Gas Control.

[7] Robeson L. M. (2008). The Upper
Bound Revisited. J. Membr. Sci..

[8] Han Y., Ho W. S. W. (2021). Polymeric
Membranes for Co2 Separation and Capture. J.
Membr. Sci..

[9] Feng S., Du X., Luo J., Zhuang Y., Wang J., Wan Y. (2023). A Review on
Facilitated Transport Membranes Based on Π-Complexation for
Carbon Dioxide Separation. Sep. Purif. Technol..

[10] Tong Z., Ho W. S. W. (2017). New Sterically
Hindered Polyvinylamine Membranes for
Co2 Separation and Capture. J. Membr. Sci..

[11] Han Y., Ho W. S. W. (2022). Mitigated Carrier
Saturation of Facilitated Transport
Membranes for Decarbonizing Dilute Co2 Sources: An Experimental and
Techno-Economic Study. J. Membrane Sci. Lett..

[12] Xu H., Pate S. G., O’Brien C. P. (2023). Mathematical
Modeling of Co2 Facilitated
Transport across Polyvinylamine Membranes with Direct Operando Observation
of Amine Carrier Saturation. Chem. Eng. J. (Lausanne).

[13] Ansaloni L., Zhao Y., Jung B. T., Ramasubramanian K., Baschetti M. G., Ho W. S. W. (2015). Facilitated Transport
Membranes Containing
Amino-Functionalized Multi-Walled Carbon Nanotubes for High-Pressure
Co2 Separations. J. Membr. Sci..

[14] Han Y., Ho W. S. W. (2018). Recent Advances
in Polymeric Membranes for Co2 Capture. Chin.
J. Chem. Eng..

[15] Pinschmidt R. K. (2010). Polyvinylamine at Last. J. Polym.
Sci. A Polym. Chem..

[16] Zhang C., Wang Z., Cai Y., Yi C., Yang D., Yuan S. (2013). Investigation of Gas Permeation Behavior
in Facilitated Transport
Membranes: Relationship between Gas Permeance and Partial Pressure. Chem. Eng. J. (Lausanne).

[17] Sigurdardottir S. B., DuChanois R. M., Epsztein R., Pinelo M., Elimelech M. (2020). Energy Barriers
to Anion Transport in Polyelectrolyte Multilayer Nanofiltration Membranes:
Role of Intra-Pore Diffusion. J. Membr. Sci..

[18] Naebe M., Wang J., Amini A., Khayyam H., Hameed N., Li L. H., Chen Y., Fox B. (2014). Mechanical Property
and Structure of Covalent Functionalised Graphene/Epoxy Nanocomposites. Sci. Rep..

[19] Shen Y., Wang H., Liu J., Zhang Y. (2015). Enhanced Performance
of a Novel Polyvinyl Amine/Chitosan/Graphene Oxide Mixed Matrix Membrane
for Co2 Capture. ACS Sustain. Chem. Eng..

[20] Wang Y., Li L., Zhang X., Li J., Liu C., Li N., Xie Z. (2019). Polyvinylamine/Graphene Oxide/Pani@Cnts Mixed Matrix Composite Membranes
with Enhanced Co2/N2 Separation Performance. J. Membr. Sci..

[21] Le L. H., Trinh D. X., Trung N. B., Tran T. P. N., Taniike T. (2017). Fabrication
of Assembled Membrane from Malonate-Functionalized Graphene and Evaluation
of Its Permeation Performance. Carbon.

[22] Bingel C. (1993). Cyclopropanierung
Von Fullerenen. Chem. Ber..

[23] Hirsch A. (1995). Addition Reactions
of Buckminsterfullerene (C60). Synthesis.

[24] Economopoulos S. P., Pagona G., Yudasaka M., Iijima S., Tagmatarchis N. (2009). Solvent-Free
Microwave-Assisted Bingel Reaction in Carbon Nanohorns. J. Mater. Chem..

[25] Economopoulos S. P., Rotas G., Miyata Y., Shinohara H., Tagmatarchis N. (2010). Exfoliation and Chemical Modification
Using Microwave
Irradiation Affording Highly Functionalized Graphene. ACS Nano.

[26] Ge B.-S., Wang T., Sun H.-X., Gao W., Zhao H.-R. (2018). Preparation
of Mixed Matrix Membranes Based on Polyimide and Aminated Graphene
Oxide for Co2 Separation. Polym. Adv. Technol..

[27] Stephenson J. J., Sadana A. K., Higginbotham A. L., Tour J. M. (2006). Highly Functionalized
and Soluble Multiwalled Carbon Nanotubes by Reductive Alkylation and
Arylation: the Billups Reaction. Chem. Mater..

[28] Stephenson J. J., Hudson J. L., Azad S., Tour J. M. (2006). Individualized Single
Walled Carbon Nanotubes from Bulk Material Using 96% Sulfuric Acid
as Solvent. Chem. Mater..

[29] Baker J. S., Kusuma V. A., Tong Z., Hopkinson D. P. (2024). Adverse
Effect of Polyelectrolyte Complexation on the Co2 Permeability of
Polyvinylamine Copolymers. ACS Appl. Eng. Mater..

[30] Kusuma V. A., McNally J. S., Baker J. S., Tong Z., Zhu L., Orme C. J., Stewart F. F., Hopkinson D. P. (2020). Cross-Linked
Polyphosphazene Blends as Robust Co2 Separation Membranes. ACS Appl. Mater. Interfaces.

[31] Deng L., Hägg M.-B. (2010). Swelling
Behavior and Gas Permeation Performance of
Pvam/Pva Blend Fsc Membrane. J. Membr. Sci..

[32] Matsuyama H., Matsui K., Kitamura Y., Maki T., Teramoto M. (1999). Effects of
Membrane Thickness and Membrane Preparation Condition on Facilitated
Transport of Co2 through Ionomer Membrane. Sep.
Purif. Technol..

[33] Kim J. H., Park S. M., Won J., Kang Y. S. (2004). Dependence
of Facilitated
Olefin Transport on the Thickness of Silver Polymer Electrolyte Membranes. J. Membr. Sci..

[34] Belaissaoui B., Lasseuguette E., Janakiram S., Deng L., Ferrari M.-C. (2020). Analysis
of Co2 Facilitation Transport Effect through a Hybrid Poly­(Allyl Amine)
Membrane: Pathways for Further Improvement. Membranes.

[35] Kuilla T., Bhadra S., Yao D., Kim N. H., Bose S., Lee J. H. (2010). Recent Advances in Graphene Based Polymer Composites. Prog. Polym. Sci..

[36] Feng X., Pelton R., Leduc M., Champ S. (2007). Colloidal Complexes
from Poly­(Vinyl Amine) and Carboxymethyl Cellulose Mixtures. Langmuir.

[37] Pelton R. (2014). Polyvinylamine:
A Tool for Engineering Interfaces. Langmuir.

[38] Guin T., Stevens B., Krecker M., D’Angelo J., Humood M., Song Y., Smith R., Polycarpou A., Grunlan J. C. (2016). Ultrastrong, Chemically Resistant
Reduced Graphene
Oxide-Based Multilayer Thin Films with Damage Detection Capability. ACS Appl. Mater. Interfaces.

[39] Venna S. R., Lartey M., Li T., Spore A., Kumar S., Nulwala H. B., Luebke D. R., Rosi N. L., Albenze E. (2015). Fabrication
of Mmms with Improved Gas Separation Properties Using Externally-Functionalized
Mof Particles. J. Mater. Chem. A.

[40] Muldoon P. F., Venna S. R., Gidley D. W., Baker J. S., Zhu L., Tong Z., Xiang F., Hopkinson D. P., Yi S., Sekizkardes A. K. (2020). Mixed Matrix Membranes
from a Microporous Polymer Blend and Nanosized Metal–Organic
Frameworks with Exceptional Co2/N2 Separation Performance. ACS Materials Lett..

[41] Feng J., Venna S. R., Hopkinson D. P. (2016). Interactions
at the Interface of
Polymer Matrix-Filler Particle Composites. Polymer.

[42] Janakiram S., Martín Espejo J. L., Yu X., Ansaloni L., Deng L. (2020). Facilitated Transport Membranes Containing
Graphene Oxide-Based Nanoplatelets
for Co2 Separation: Effect of 2d Filler Properties. J. Membr. Sci..

[43] Xu W., Lindbråthen A., Janakiram S., Ansaloni L., Deng L. (2023). Enhanced Co2/H2
Separation by Go and Pva-Go Embedded Pvam Nanocomposite Membranes. J. Membr. Sci..

[44] Casadei R., Venturi D., Giacinti Baschetti M., Giorgini L., Maccaferri E., Ligi S. (2019). Polyvinylamine Membranes
Containing Graphene-Based Nanofillers for
Carbon Capture Applications. Membranes.

[45] Li Y., Dekel D. R., He X. (2024). Carrier-Driving
Co2 Separation by
Amine-Rich Membranes with Intercalated Graphene Oxide. J. Membr. Sci..

[46] Kim T.-J., Vrålstad H., Sandru M., Hägg M.-B. (2013). Separation
Performance of Pvam Composite Membrane for Co2 Capture at Various
Ph Levels. J. Membr. Sci..

[47] Han Y., Wu D., Ho W. S. W. (2019). Simultaneous
Effects of Temperature and Vacuum and
Feed Pressures on Facilitated Transport Membrane for Co2/N2 Separation. J. Membr. Sci..

[48] Deng L., Hägg M.-B. (2014). Carbon Nanotube Reinforced Pvam/Pva
Blend Fsc Nanocomposite
Membrane for Co2/Ch4 Separation. Int. J. Greenh.
Gas Control.

[49] Han Y., Wu D., Ho W. S. W. (2018). Nanotube-Reinforced Facilitated Transport Membrane
for Co2/N2 Separation with Vacuum Operation. J. Membr. Sci..

[50] Chen K. K., Han Y., Zhang Z., Ho W. S. W. (2021). Enhancing
Membrane Performance for
Co2 Capture from Flue Gas with Ultrahigh Mw Polyvinylamine. J. Membr. Sci..

[51] Lai, T.-W.; Vijayendran, B. R. Acidized fracturing fluids containing high molecular weight poly(vinylamines) for enhanced oil recover. US Patent 4843118A, University of Pittsburgh, 1989.

[52] Gu L., Zhu S., Hrymak A. N. (2002). Acidic and Basic Hydrolysis of Poly­(N-Vinylformamide). J. Appl. Polym. Sci..

