# Supplementary material for: Mixed Matrix Membranes Composed of Graphene-Based Derivatives as Additives in PVAm for CO2 Capture
Source: ACS Appl Eng Mater. 2025 Aug 6;3(8):2430–40. doi: 10.1021/acsaenm.5c00316 (PMC12379166; doi:10.1021/acsaenm.5c00316)
Supplement: Supplementary file 1 [file em5c00316_si_001.pdf]

Supporting Information

Mixed matrix membranes composed of graphene-based derivatives as additives in PVAm for CO_2 capture

James Baker, [a,b] *Cristina Perinu,* [c] *Maria Psarrou,* [c] *Sigmund Mordal Lucasen,* [c] *Victor Kusuma,* [a,b] *Patrick F. Muldoon,* [a,b] *Akrivi Asimakopoulou,* [d] *David Hopkinson,* [a] *Solon P. Economopoulos**[c]

[a] U.S. Department of Energy, National Energy Technology Laboratory, 626 Cochrans Mill Rd, Pittsburgh, Pennsylvania 15236, USA

[b] NETL Support Contractor, 626 Cochran Mill Rd, Pittsburgh, PA 15236, USA

[c] Department of Chemistry, Norwegian University of Science and Technology, 7491 Trondheim, Norway

[d] Advanced Renewable Technologies & Environmental Materials in Integrated Systems, ARTEMIS, Chemical Process and Energy Resources Institute CPERI, Centre for Research and Technology Hellas CERTH, Thermi, 57001 Thessaloniki, Greece

Corresponding Author email: solon.oikonomopoulos@ntnu.no

The supporting information document contains images of polymer-graphene dispersions, additional storage moduli at 30 °C, chemical structure of PVAm used in this work and Robeson plot for all PVAm-based bulk films as discussed in the main text.

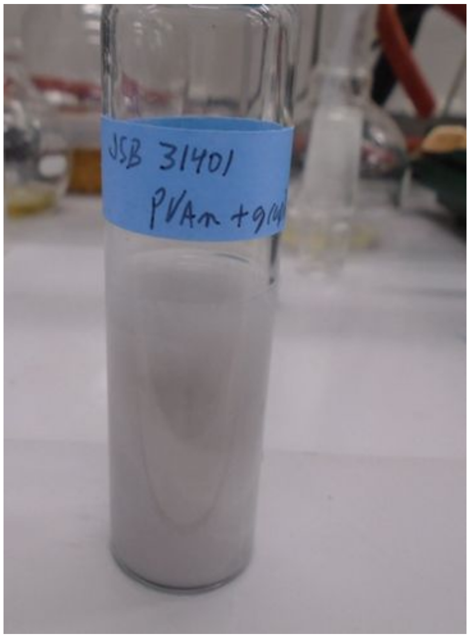

Figure S1. Solution of 0.66 wt% **PVAm** in H_2O with **G-TEG** added as a 1 mg/mL suspension in benzyl alcohol (BzOH). **G-TEG** content is 0.5 wt% relative to **PVAm**.

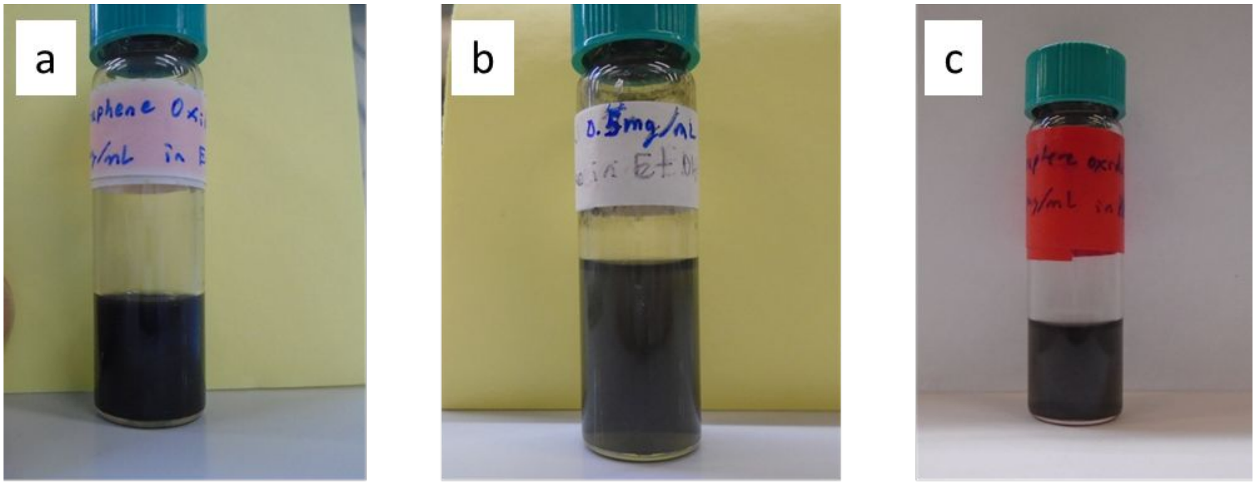

Figure S2. Dispersions of a) commercial graphene oxide (**GO**) and b) **G-TEG** at 0.5 mg/mL in 1:1 EtOH:H_2O. c) **GO-EDAOH** 0.2 mg/mL in MeOH after sonication

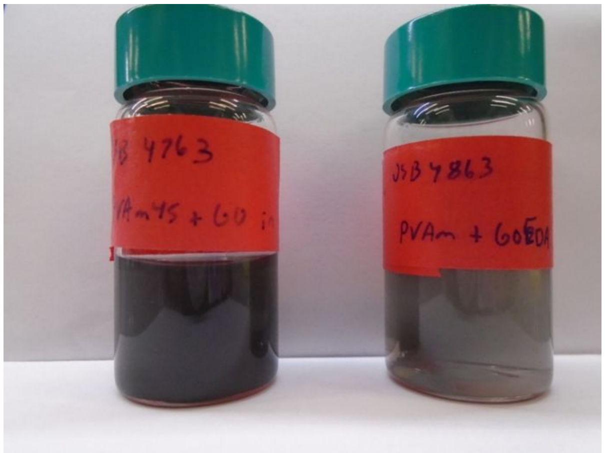

Figure S3. Dispersions of 1 wt% **PVAm** in H_2O containing 0.05 mg/mL (0.5 wt% relative to **PVAm**) commercial graphene oxide (left) and **GO-EDAOH** (right)

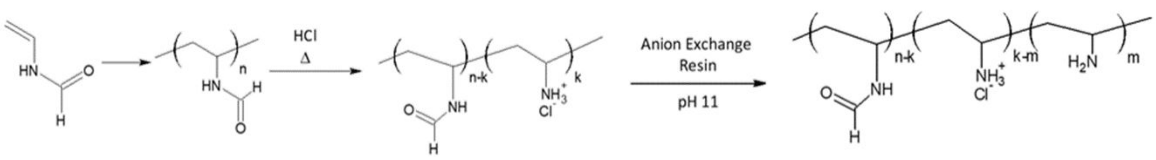

Figure S4. Chemical structure of poly(vinylamine-co-N-vinylformamide) (**PVAm**)

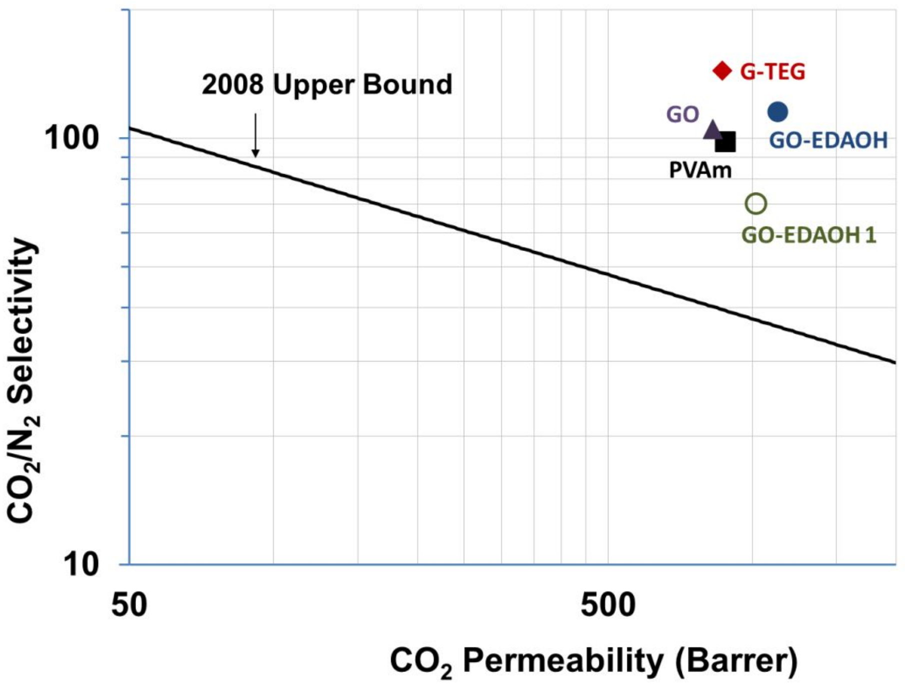

Figure S5. Robeson plot for PVAm bulk films containing 0.5 wt% nanofiller (except **GO-EDAOH 1** contains 1 wt% filler) relative to PVAm. Testing conditions: 60 ˚C, 95% relative humidity, feed gas 70 sccm 4/96 CO_2/N_2 at 1.5 ata, sweep gas 20 sccm He at 1.22 ata.

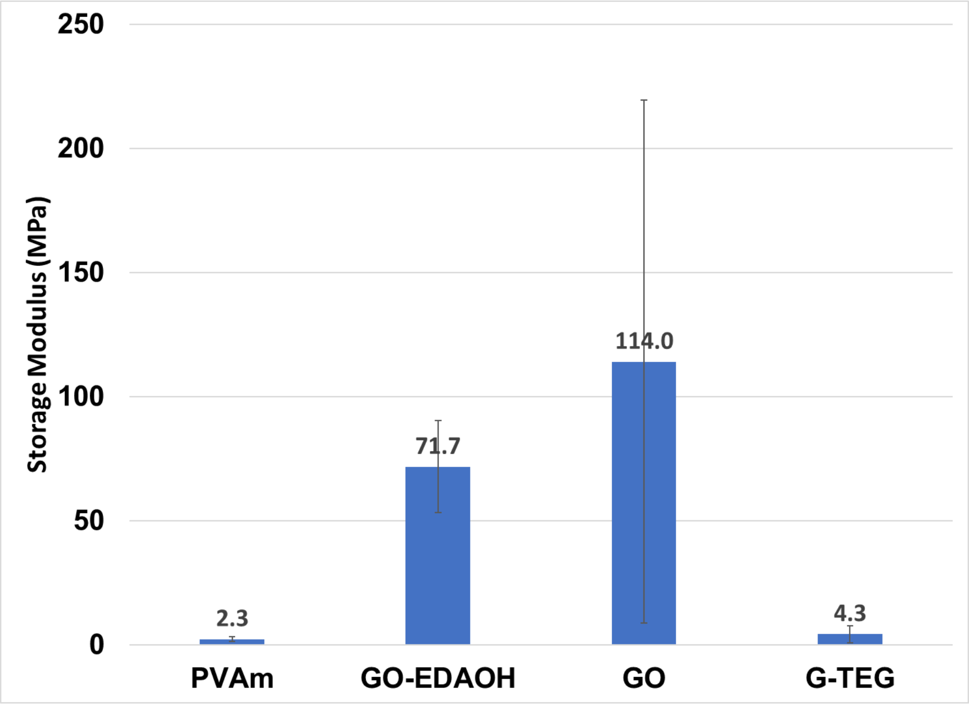

Figure S6. Comparison of the storage modulus for PVAm films under 50 % relative humidity N_2 atmosphere at 30 ˚C as measured by DMA-RH.
